# Nasogastric tube‐administered alectinib achieved long‐term survival in a crizotinib‐refractory nonsmall cell lung cancer patient with a poor performance status

**DOI:** 10.1002/ccr3.973

**Published:** 2017-04-26

**Authors:** Osamu Kanai, Young Hak Kim, Koichi Nakatani, Kohei Fujita, Tadashi Mio

**Affiliations:** ^1^ Division of Respiratory MedicineNational Hospital Organization Kyoto Medical CenterKyotoJapan; ^2^ Department of Respiratory MedicineGraduate School of MedicineKyoto UniversityKyotoJapan

**Keywords:** Alectinib, anaplastic lymphoma kinase, leptomeningeal carcinomatosis, nasogastric tube, nonsmall cell lung cancer

## Abstract

Alectinib shows remarkable efficacy against anaplastic lymphoma kinase (ALK)‐positive nonsmall cell lung cancer (NSCLC), with minimal adverse effects. Therefore, alectinib may provide a survival benefit to ALK‐positive NSCLC patients with a poor performance status. If the medication cannot be taken by mouth, the patient may be given alectinib through a nasogastric tube.

## Introduction

Cytotoxic chemotherapy is the standard therapy for patients with advanced nonsmall cell lung cancer (NSCLC). Recently, several molecular targeted therapies have demonstrated promising efficacy against NSCLC. To date, these targeted treatments are routinely performed as personalized therapies for patients with NSCLC whose tumors harbor driver oncogene mutations, such as an epidermal growth factor receptor (EGFR) mutation or a fusion of echinoderm microtubule‐associated protein‐like 4 (EML4) with anaplastic lymphoma kinase (ALK). A gefitinib treatment study suggests that molecular targeted therapies would be effective and well tolerated in patients with a poor performance status (PS), which is equivalent to an Eastern Cooperative Oncology Group PS (ECOG‐PS) score of two or higher 1. Alectinib, a selective ALK inhibitor, has shown clinical activity and favorable tolerability in ALK inhibitor‐naïve and crizotinib‐refractory NSCLCs with the ALK rearrangement 2, 3, 4. However, whether alectinib provides a benefit to the ALK‐positive NSCLC patient with a poor PS has not been confirmed. Here, we report the achievement of a postdisease progression survival time that exceeded 14 months when a crizotinib‐refractory ALK‐positive NSCLC patient with a poor PS was administered alectinib through a nasogastric tube.

## Case Presentation

**Figure 1:**
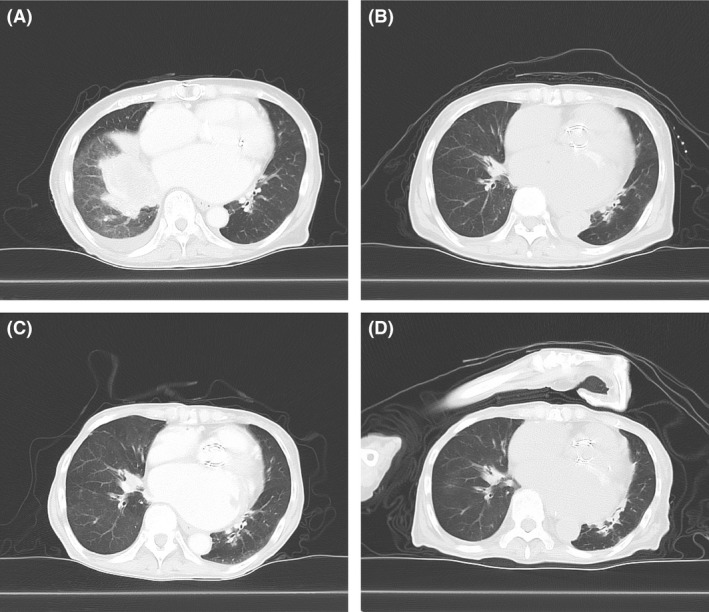
Computed tomography (CT) images of the right lower lobe of the lung. A and B show CT images obtained at 5 days before initiation of crizotinib and 1 month before discontinuation, respectively. C and D show CT images obtained at 2 weeks before and 6 months after initiation of alectinib, respectively.

**Figure 2:**
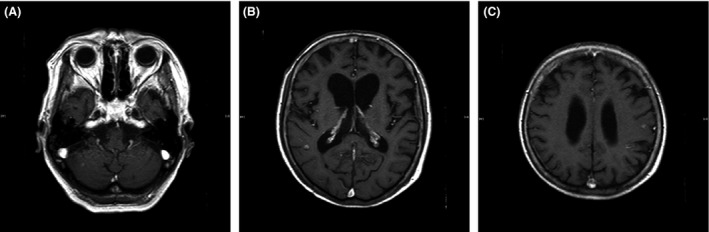
Enhanced brain magnetic resonance imaging (MRI) images taken before withdrawal of crizotinib. A shows a newly appeared dissemination on the right cerebellar surface. B and C show pre‐existing cerebral tumors in the right and left temporal lobes, respectively.

A 76‐year‐old woman presented with bloody sputum. Chest computed tomography (CT) showed both a huge mass with atelectasis in the right lower lobe of the lung and bilateral adrenal tumors (Fig. 1A). A CT‐guided percutaneous lung biopsy confirmed the diagnosis of lung adenocarcinoma. However, symptoms of motor aphasia, nausea, and walking difficulty rapidly developed, and the patient's ECOG‐PS decreased from 1 to 3. The enhanced brain CT revealed multiple brain metastases that presumably caused her symptoms. Whole‐brain radiation therapy (30 Gy/10 fractions) was immediately performed. Additionally, administration of crizotinib (250 mg, twice daily) was initiated after radiation therapy because the patient's tissue samples demonstrated EML4‐ALK fusion oncogenes according to immunohistochemistry and a fluorescence in situ hybridization assay. Although nausea and colitis required a crizotinib reduction to one daily 250‐mg dose within 1 month, dramatic tumor shrinkage and an improvement in the patient's ECOG‐PS score to one were achieved (Fig. 1B). One year and four months later, she developed somnolence and dysphagia. Brain magnetic resonance imaging (MRI) revealed new disseminated tumors on the brain surface, suggesting leptomeningeal carcinomatosis (LC) (Fig. 2A–C). Because the newly developed neurological syndrome decreased the patient's PS and ability to tolerate an invasive procedure, a lumbar puncture was not performed. Pemetrexed (400 mg/m^2^), which was expected to be the most tolerable cytotoxic agent used in the NSCLC treatment, was administered after crizotinib was discontinued. However, severe general malaise increased the patient's ECOG‐PS score to four and necessitated the discontinuation of the pemetrexed treatment after the first course. A shift from chemotherapy to palliative care for the patient was considered, but chemotherapy was continued at the behest of her family. Three months after the pemetrexed treatment, alectinib became commercially available in Japan, and a 300‐mg twice daily dose of alectinib was initiated. Because of the LC‐associated somnolence and dysphagia, a nasogastric tube was placed to administer alectinib and the infusing nutrients. A modest response was shown on CT 6 months later, but the patient survived for several months without any adverse effects (AE) (Fig. 1C and D). Except for somnolence, the patient's neurological symptoms remained unaffected. However, administration of alectinib through a nasogastric tube enabled the patient to live with her family at home for over 14 months from the initiation of the alectinib treatment.

## Discussion

Here, we report the safe administration of alectinib for over 14 months to an ALK‐positive NSCLC patient with a poor PS after failed crizotinib therapy. Alectinib caused no AEs, but crizotinib required a dose reduction due to nausea and colitis. Moreover, the patient's PS score increased due to pemetrexed‐associated general malaise. Alectinib was previously reported to induce complete remission in a patient with NSCLC whose LC had progressed, while the patient was on crizotinib 5. Nevertheless, the patient in our study survived for over 14 months due to the alectinib treatment, even though her PS was remarkably worse than that of the aforementioned case report. Compared to the median survival time for NSCLC patients with LC (14 weeks), our patient achieved a remarkably long survival time (measured from LC onset) with the alectinib treatment 6. This survival benefit likely resulted from the impressive overall response rate (ORR), response time, and minimal AEs that have been observed in crizotinib‐refractory ALK‐positive NSCLC patients with central nervous system metastases 3, 4, 7. The most common alectinib‐associated symptomatic AEs were constipation (36%), fatigue (33%), and myalgia (24%), whereas those for crizotinib were vision disorder (71%), diarrhea (61%), and nausea (56%). Pemetrexed‐associated AEs included fatigue (34%), nausea (31%), and vomiting (16%) 4, 8, 9. Like gefitinib, alectinib is expected to provide a therapeutic benefit to ALK‐positive NSCLC patients with poor PS scores due to its high ORR and minimal AEs 1.

The nasogastric tube was a useful tool for the safe administration of alectinib to our patient, who could not swallow the oral capsule. The nasogastric tube was used to administer alectinib to the patient for three specific reasons. First, somnolence and dysphagia rendered the patient incapable of swallowing the capsule. Second, oral alectinib would have required the patient to swallow eight capsules twice daily, which would have been more difficult than the oral crizotinib treatment. Third, the patient's poor PS suggested that she would be intolerant to a percutaneous endoscopic gastrostomy. Similar reports have described the administration of molecular targeted agents through nasogastric tubes, including the administration of crizotinib to a patient with gastrointestinal toxicities 8, erlotinib to a critically ill patient who required mechanical ventilation 9, and erlotinib to a patient with an LC‐derived consciousness disorder 10. Administration through a nasogastric tube is an alternative method for providing the therapeutic benefit of alectinib to a patient with NSCLC who cannot swallow the capsule.

The alectinib treatment (300 mg twice daily) did not sufficiently improve the neurological symptoms of the patient. We did not increase the alectinib dose because the maximum dose had been determined by the Ministry of Health, Labour and Welfare in Japan to be 300 mg twice daily, while the recommended dose for alectinib was determined to be 600 mg twice daily in a phase II study 7. Considering the linear correlation between the concentrations of free alectinib in the cerebrospinal fluid and in the serum, the dose escalation of alectinib to two daily doses of 600 mg or more might improve the patient's neurological symptoms 7.

## Conclusion

Alectinib may provide a survival benefit to ALK‐positive NSCLC patients with a poor PS, even when crizotinib treatment fails. Considering the expected magnitude of efficacy, the administration of alectinib through a nasogastric tube can be offered to ALK‐positive patients who cannot swallow medication. Further investigations are warranted to determine the effect of alectinib in NSCLC patients with a poor PS.

## Authorship

OK: wrote and revised the manuscript. YHK: designed and revised the manuscript. KN: treated the patient and conceived the idea. KF: designed and revised the manuscript. TM: conceived the idea and designed the manuscript.

## Conflicts of Interest

None declared.
